# Mutation in *Fbxo11* Leads to Altered Immune Cell Content in *Jeff* Mouse Model of Otitis Media

**DOI:** 10.3389/fgene.2020.00050

**Published:** 2020-02-11

**Authors:** Pratik P. Vikhe, Hilda Tateossian, Gurpreet Bharj, Steve D.M. Brown, Derek W. Hood

**Affiliations:** Mammalian Genetics Unit, MRC Harwell Institute, Oxfordshire, United Kingdom

**Keywords:** *Fbxo11*, otitis media, *Jeff* mouse, immune cells, natural killer cells

## Abstract

The *Jeff* mouse mutant carries a mutation in the F-box only 11 gene (*Fbxo11*) and heterozygous animals display conductive deafness due to the development of otitis media (OM). The *Fbxo11* locus is also associated with chronic otitis media with effusion (COME) and recurrent OM in humans. The *Jeff* mutation affects the ability of FBXO11 to stabilize p53 that leads to perturbation in the TGF-beta/Smad2 signaling pathway important in immunity and inflammation. In the current study, we evaluated the effect of the *Jeff* mutation on the immune cell content using multicolor flow cytometry. In blood of *Jeff* heterozygotes, we observed a significant increase in the number of NK, dendritic (CD11b+), neutrophils, and natural killer T (NKT) cells and a significant decrease in effector T-helper and B-lymphocytes compared to wild-type controls. The percentage of NK cells significantly decreased in the lungs of *Jeff* heterozygotes, with a concomitant reduction in B-lymphocytes and T-cytotoxic cells. In the spleen, *Jeff* heterozygotes displayed a significant decrease in mature B-lymphocytes, effector T-helper, and naïve T-cytotoxic cells. Neutrophils, dendritic, and NKT cells dominated bulla fluid in *Jeff* heterozygote mice. Similar analysis carried out on *Fbxo11^tm2b/+^* heterozygotes, which carry a null allele, showed no difference when compared to wild-type. Cytokine/chemokine analysis revealed a significant increase in the G-CSF, GM-CSF, sTNFRI, TPO, and IL-7 levels in *Jeff* heterozygote serum compared to wild-type. This analysis increases our understanding of the role played by *Fbxo11*, a gene associated with human OM, in the systemic and localized cellular immune response associated with increased susceptibility to OM.

## Introduction

Otitis media (OM) is a very common middle ear disease in children that is characterized by middle ear inflammation and fluid accumulation (Schilder et al., 2016). Pathology of OM can manifest in different ways and chronic and recurrent OM (ROM), often accompanied by effusion (chronic OM with effusion, COME), can lead to hearing loss and developmental delays with a significant burden on the healthcare system (Veenhoven et al., 2003). Tympanostomy to alleviate COME remains the commonest cause of surgery in children. There is a significant genetic component to COME (Sale et al., 2011), and recently the discovery of a number of mouse genetic models of COME has led to insights into the genetic and molecular pathways involved in the pathophysiology of chronic middle ear disease (Li et al., 2013; Bhutta et al., 2017b). *Jeff* (Hardisty et al., 2003), *Junbo* (Parkinson et al., 2006), and *edison* (Crompton et al., 2017) mutants are COME mouse models each with single point mutation in the *Fbxo11, Evi1,* and *nischarin* genes respectively, identified at MRC Harwell through a deafness screen as a part of a larger scale N-ethyl-N-nitrosourea (ENU) mouse mutagenesis program (Nolan et al., 2000).

Following the identification of a number of genes involved in COME in the mouse there have been several studies to explore the association of these genes with COME in the human population. Association studies have shown that several human genes may increase susceptibility toward OM, including *FBXO11* (Sale et al., 2011). One of the largest association studies employing samples from 1,296 families (3,828 individuals) uncovered a significant link between FBXO11 and OM susceptibility (Bhutta et al., 2017a). Moreover, two similar studies carried out on western Australian children (Rye et al., 2011) and Minnesota COME/ROM families (Segade et al., 2006) showed a direct link between polymorphisms at the *FBXO11* locus and OM susceptibility.

FBXO11 is part of the SCF (SKP1-cullin-F-box) complex, a multi-protein E3 ubiquitin-ligase complex catalyzing the ubiquitination of proteins destined for proteasomal degradation. FBXO11 is involved in ubiquitination of BCL6 (Duan et al., 2012) and phosphorylated SNAIL (Zheng et al., 2014), and neddylation of p53 (Abida et al., 2007), which play critical roles in regulation of the mammalian cell cycle and function. FBXO11, through regulation of BCL6, modulates B-cell survival and plays a crucial role in B-cell lymphoma (Duan et al., 2012). Other than regulation of B-cell survival in lymphoma, the role played by FBXO11 in modulating other immune cells and the relevance of this to OM remains unclear.

In the current study, we used the well-characterized COME *Jeff* mouse model (Hardisty et al., 2003; Hardisty-Hughes et al., 2006) which carries a missense mutation in the *Fbxo11* gene. The homozygotes of this mutant mouse line do not survive beyond birth and show a severe lung phenotype (Tateossian et al., 2009), whereas the heterozygote mice survive with chronic OM (Hardisty et al., 2003). FBXO11 can function as a Nedd8-ligase for p53 promoting its neddylation and inhibiting its transcription activity. The *Fbxo11* mutation in the *Jeff* mouse results in a decreased level of p53 and increased pSmad2 levels; pSmad2 is directly involved in the regulation of the TGF-beta pathway (Tateossian et al., 2009; Tateossian et al., 2015). The TGF-beta pathway plays a critical role in maintaining homeostasis by regulating remodeling of injury in disease and modulating the immune and inflammatory response (Wan and Flavell, 2007).

The association of *FBXO11* locus polymorphisms with OM in the human population and the corresponding phenotype observed in the *Jeff* mouse with a mutation in the orthologous gene makes it a vital model to dissect important immune changes that are associated with OM. In the current study, we analyzed the cellular immune changes that occur due to the *Fbxo11* mutation in the heterozygote *Jeff* mouse using a comprehensive flow cytometry panel that classified granulocytes, monocytes, macrophages, eosinophils, dendritic, natural killer, B and T cells. The *Jeff* mutation significantly altered the immune cell content in the blood, mostly affecting the natural killer cells, and adaptive B and T immune cells, which underlines the significance of FBXO11 in the immune/genetic regulation of these cells. Further, we carried out a similar analysis with the heterozygote *Fbxo11* knockout mouse in order to better understand the role of FBXO11 in the cellular immune response. The *Fbxo11* knockout mouse did not show significant differences in the immune cells as was observed in the *Jeff* mouse, suggesting that the *Fbxo11* mutation in the *Jeff* mouse has gain of function effects that lead to altered immune cell content. Also, using a mouse model, this study is the first to report systemic immune cell differences due to a genetic aberration leading to localized middle ear inflammation and OM.

## Materials and Methods

### Mice

The humane care and use of mice in this study was carried out under the appropriate UK Home Office license and the local ethics review committee reviewed the experimental procedures.

Heterozygote *Fbxo11^Jf/+^* (also referred to as *Jeff* Het) mice and their *Jeff* wild-type littermates (also referred to as *Jeff* Wt) were generated by inter-crossing F1 *Fbxo11^Jf/+^* C57BL/6J C3H/HeH males with F1 *Fbxo11^+/+^* C57BL/6J C3H/HeH wild-type females. Heterozygote *Fbxo11^tm2b/+^* (also referred to as *Fbxo11* knockout) and their *Fbxo11^+/+^* wild-type littermates were generated by inter-crossing F1 *Fbxo11^tm2b/+^*-PL-TM2B-C57BL/6NJ with C57BL/6NJ females. The mice were specific pathogen free and used at 8 weeks of age.

### Sample Preparation

Blood was collected by retro-orbital bleeding under terminal anesthesia induced by an intraperitoneal overdose of sodium pentobarbital in lithium heparin tubes. For flow cytometry analysis, 50 µl of blood was resuspended in 1 ml of red blood cell (RBC) lysis buffer (Biolegend™) for 10 min on ice followed by centrifugation and two washes with 1 ml phosphate buffer saline (PBS). The final pellet was resuspended in FACS buffer (5 mM EDTA, 0.5% fetal calf serum in PBS). Middle ear fluid for flow cytometry and cytokine/chemokine analyses was collected as described previously (Vikhe et al., 2019). Briefly, middle ear fluid was collected from unskinned heads obtained from mice under terminal anesthesia. After removal of any material on the external surface of the tympanic membrane, a hole was made in the membrane while removing the malleus from the middle ear using a sterile pair of forceps and fluid was collected with a pipette and microtip in 10 μl cold PBS. Other tissues (spleens and lungs) were collected in gentleMACS™ C tubes with 3 ml RPMI supplemented with 10 µg/ml collagenase II (Serlabo™) and 166 µg/ml DNAse I (Sigma™). The tissue was homogenized in a gentleMACS™ Dissociator and the single cell suspension was separated from tissue debris by passing through a 70 µm Cell Strainer. The cell pellet obtained after centrifugation at 800 x g for 2 min was resuspended in RBC lysis buffer. Finally, after RBC lysis, the cells were pelleted by centrifugation then resuspended in FACS buffer for flow cytometry analysis.

### Flow Cytometry Analysis

Middle ear fluid, blood, lung, and spleen samples were diluted with FACS buffer, and a cell count was obtained. The samples were transferred to a 96 well plate at the concentration of 2 X 10^5^ cells/well. Cells were centrifuged and washed twice with FACS buffer. For the analysis of different types of immune cells, the resuspended cells were incubated for 15 min with CD16/CD32 antibody (BD Pharmagen™) at a dilution of 1:100. After centrifugation at 800 x g for 1 min, cell pellets were resuspended in 100 µl of either of panel 1 or panel 2 (**Table S1**) antibody cocktail and incubated for 20 min in the dark. Cells were centrifuged and washed twice with FACS buffer and finally resuspended in 100 µl of Sytox DNA stain (1:10,000). After making up the final volume to 250 µl with FACS buffer, flow cytometry was performed using a BD FACSCanto™ II system. FlowJo software (Tree Star™) was used to analyze the data obtained (**Figures S1**, **S2** and **Table S2**).

### Cytokine/Chemokine Analysis

Cytokine and chemokine levels were measured using a mouse cytokine antibody array (Mouse Cytokine Array C1, RayBiotech Inc.™) as described previously (Vikhe et al., 2019). Changes in IL-7 and IL-15 were analyzed by Mouse IL-7 and IL-15 DuoSet ELISA (R&D systems™) according to the manufacturer's instructions. The color change was measured at 450 nm on an Epoch BioTek™ plate reader and the IL-7 and IL-15 concentrations were calculated by using the standard curve obtained.

### Data Analysis

We used the unpaired t test assuming equal variance for comparing the cytokine/chemokine changes between *Fbxo11^Jf/+^* serum to the *Jeff* wild-type whereas non-parametric Mann-Whitney U test was used to compare the flow cytometry data. A *P* value of less than 0.05 was considered significant.

## Results

### Innate Immune Cell Population in *Fbxo11^Jf/+^* Mouse

**Figure 1:**
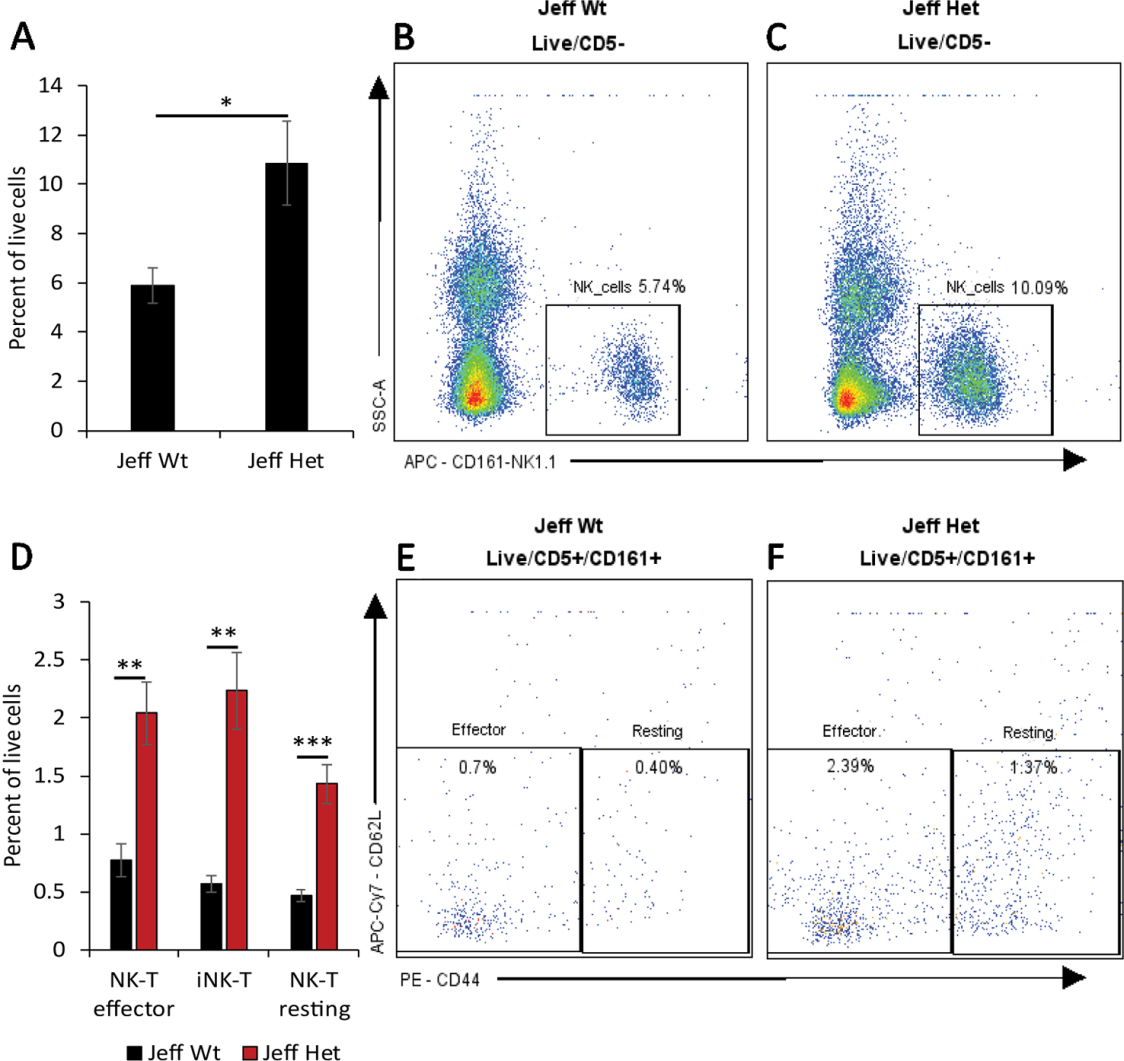
Natural killer (NK) cell percentage in *Jeff* mice (*Fbxo11^Jf/+^* and *Jeff* Wt). **(A)** Average difference in the percentage of blood NK cells in *Jeff* Het (*Fbxo11^Jf/+^*) and *Jeff* wild type (*Jeff* Wt) mice. The error bars show standard error of mean where n = 4 and *P < 0.05. Representative flow cytometry plot for NK cell analysis of bloods from *Jeff* Wt **(B)** and *Jeff* Het (*Fbxo11^Jf/+^*) **(C)** mice. **(D)** Average difference in the percentage of blood effector NKT, resting NKT, and invariant NKT (iNKT) cells in *Jeff* Het (*Fbxo11^Jf/+^*) and *Jeff* Wt mice. The error bars show standard error of mean where n = 4 and *P < 0.05, **P < 0.01, ***P < 0.001. Representative flow cytometry plot for NKT cell analysis of bloods from *Jeff* Wt **(E)** and *Jeff* Het (*Fbxo11^Jf/+^*) **(F)** mice.

Innate immune cells play a critical role in the initiation of the inflammatory response and the onset and maintenance of OM (Mittal et al., 2014). The *Fbxo11^Jf/+^* mouse is characterized by middle ear inflammation that mimics the human OM and also exhibits a defective lung phenotype (Tateossian et al., 2009). To understand the effect of the *Jeff* mutation in the *Fbxo11* gene on the innate immune cell population we analyzed the neutrophils, eosinophils, macrophages, monocytes, dendritic cells (DC) (CD11b+ve and –ve), progenitor DC, and natural killer (NK) cells present in blood, spleen, lungs, and middle ear fluids obtained from the *Fbxo11^Jf/+^* mouse (**Table 1**). Significant differences were observed in the proportion of blood neutrophils, macrophages, DCs, and NK cells in *Fbxo11^Jf/+^* when compared to the *Jeff* wild-type litter mates. The greatest difference was observed in blood NK cell levels, which were 10.84% of live cells in the *Fbxo11^Jf/+^* mouse compared to 5.87% in the *Jeff* wild-type counterpart (**Figure 1A–C**). The *Fbxo11^Jf/+^* mouse had 3.31% of CD11b+ve DCs compared to 1.29% present in *Jeff* wild-type blood. Similarly, slight but significantly higher levels of macrophages (0.57%) were observed in *Fbxo11^Jf/+^* compared to 0.20% in *Jeff* wild-type blood. Blood granulocyte levels were significantly higher in the *Fbxo11^Jf/+^* (19.30%) compared to *Jeff* wild-type (16.60%) mouse. In contrast to blood, a significantly lower proportion of NK cells was observed in lungs of *Fbxo11^Jf/+^* (0.91%) compared to *Jeff* wild-type (2.80%) mice. Further, *Fbxo11^Jf/+^* lungs had 0.81% of monocytes compared to 1.58% in the *Jeff* wild-type mouse. No significant difference was observed in *Fbxo11^Jf/+^* spleen innate immune cells when compared to the *Jeff* wild-type mouse. The middle ear fluid from *Fbxo11^Jf/+^* had a high percentage of granulocytes and DCs. The middle ear fluid comprised 10.34% neutrophils and 6.69% progenitor dendritic cells (pDCs). Compared to pDC's, the percentage of CD11b-ve DCs and CD11b+ DCs in the *Fbxo11^Jf/+^* middle ear fluid was less: 3.69 and 4.54% respectively (**Table 1**).

**Table 1:** Immune cell percentage in *Jeff* wild-type (*Jeff* Wt) and *Fbxo11^Jf/+^* (*Jeff* Het) mouse obtained by flow cytometry.

Immune cells	Blood		Lungs		Spleen		MEF
	*Jeff* Wt	*Jeff* Het	*Jeff* Wt	*Jeff* Het	*Jeff* Wt	*Jeff* Het	*Jeff* Het
**Granulocytes**	**16.60 ( ± 1.76)**	**19.30 ( ± 0.85)**	6.21 ( ± 0.79)	5.34 ( ± 0.43)	3.53 ( ± 0.43)	2.77 ( ± 0.31)	10.34 ( ± 4.11)
**Eosinophils**	0.18 ( ± 0.07)	0.29 ( ± 0.05)	0.035 ( ± 0.01)	0.028 ( ± 0.01)	0.05 ( ± 0.03)	0.021 ( ± 0.01)	0.14 ( ± 0.05)
**Macrophages**	**0.20 ( ± 0.06)**	**0.57 ( ± 0.16)**	0.21 ( ± 0.04)	0.22 ( ± 0.03)	0.34 ( ± 0.04)	0.23 ( ± 0.02)	0.22 ( ± 0.06)
**Monocytes**	5.54 ( ± 0.88)	8.41 ( ± 1.55)	**1.58 ( ± 0.21)**	**0.81 ( ± 0.22)**	0.89 ( ± 0.03)	0.84 ( ± 0.12)	0.34 ( ± 0.16)
**DC CD8 type**	27.5 ( ± 2.78)	20.44 ( ± 2.19)	13.52 ( ± 1.53)	13.17 ( ± 1.43)	43.60 ( ± 0.70)	42.30 ( ± 1.58)	3.69 ( ± 0.72)
**DC CD11b type**	**1.29 ( ± 0.28)**	**3.31 ( ± 0.53)**	1.06 ( ± 0.15)	1.35 ( ± 0.13)	1.81 ( ± 0.17)	1.92 ( ± 0.11)	4.54 ( ± 0.99)
**Progenitor DC (pDC)**	7.54 ( ± 1.96)	7.99 ( ± 1.06)	10.65 ( ± 0.89)	11.13 ( ± 1.36)	5.43 ( ± 0.75)	4.50 ( ± 0.43)	6.69 ( ± 1.46)
**NK cells**	**5.87 ( ± 0.72)**	**10.84 ( ± 1.71)**	2.80 ( ± 0.17)	0.91 ( ± 0.23)	4.09 ( ± 0.63)	3.12 ( ± 0.20)	0.79 ( ± 0.35)
**T helper (Th) effector**	**2.63 ( ± 0.72)**	**0.69 ( ± 0.03)**	0.37 ( ± 0.13)	0.17475 ( ± 0.12)	**1.28 ( ± 0.06)**	**0.72 ( ± 0.01)**	0.22 ( ± 0.07)
**T helper (Th) resting**	4.49 ( ± 0.59)	4.54 ( ± 0.68)	2.45 ( ± 0.50)	1.22 ( ± 0.32)	7.73 ( ± 0.71)	8.93 ( ± 0.61)	0.16 ( ± 0.07)
**T regulator (T reg) effector**	0.86 ( ± 0.08)	0.66 ( ± 0.06)	0.68 ( ± 0.12)	0.52 ( ± 0.12)	0.53 ( ± 0.05)	0.46 ( ± 0.03)	0.28 ( ± 0.08)
**T regulator (T reg) resting**	**0.14 ( ± 0.02)**	**0.43 ( ± 0.06)**	0.35 ( ± 0.06)	0.22 ( ± 0.07)	**0.82 ( ± 0.01)**	**1.34 ( ± 0.09)**	0.029 ( ± 0.01)
**T cytotoxic effector**	0.47 ( ± 0.09)	0.31 ( ± 0.01)	0.31 ( ± 0.10)	0.11 ( ± 0.03)	0.53 ( ± 0.08)	0.34 ( ± 0.03)	0.68 ( ± 0.27)
**T cytotoxic naive**	3.62 ( ± 0.67)	2.53 ( ± 0.38)	**1.25 ( ± 0.12)**	**0.58 ( ± 0.12)**	5.56 ( ± 0.73)	6.92 ( ± 0.72)	0.07 ( ± 0.04)
**T cytotoxic resting**	**1.56 ( ± 0.35)**	**0.67 ( ± 0.12)**	**0.44 ( ± 0.07)**	**0.10 ( ± 0.03)**	**2.44 ( ± 0.25)**	**1.64 ( ± 0.17)**	0.04 ( ± 0.02)
**B2 total**	**24.82 ( ± 2.80)**	**11.16 ( ± 1.22)**	11.53 ( ± 1.09)	9.22 ( ± 0.91)	34.90 ( ± 1.07)	28.87 ( ± 0.95)	1.37 ( ± 0.34)
**B2 mature**	**2.31 ( ± 0.25)**	**1.55 ( ± 0.09)**	2.69 ( ± 0.48)	3.75 ( ± 0.27)	3.38 ( ± 0.28)	2.97 ( ± 0.09)	0.26 ( ± 0.11)
**B2 immature**	**21.98 ( ± 2.79)**	**9.26 ( ± 1.13)**	**8.52 ( ± 0.85)**	**5.14 ( ± 0.70)**	30.67 ( ± 0.91)	25.20 ( ± 0.83)	1.03 ( ± 0.23)
**B1 total**	**0.73 ( ± 0.09)**	**0.58 ( ± 0.03)**	**3.14 ( ± 0.48)**	**5.81 ( ± 0.76)**	3.43 ( ± 0.38)	4.90 ( ± 0.44)	1.74 ( ± 0.32)
**NKT effector**	**0.77 ( ± 0.14)**	**2.04 ( ± 0.27)**	12.92 ( ± 0.88)	16.22 ( ± 2.22)	1.29 ( ± 0.18)	0.95 ( ± 0.07)	3.15 ( ± 0.99)
**Invariant NKT (iNKTs)**	**0.57 ( ± 0.07)**	**2.23 ( ± 0.33)**	6.21 ( ± 1.09)	8.98 ( ± 1.40)	1.13 ( ± 0.24)	1.47 ( ± 0.15)	1.99 ( ± 0.67)
**NKT resting**	**0.46 ( ± 0.05)**	**1.43 ( ± 0.16)**	**0.96 ( ± 0.11)**	**1.43 ( ± 0.35)**	**0.78 ( ± 0.11)**	**1.63 ( ± 0.20)**	0.68 ( ± 0.17)

Values are the percentage of total live cells. Values in blue are significantly increased whereas those in red are significantly decreased in Jeff Het (Fbxo11^Jf/+^) compared to Jeff Wt (Jeff wild-type) mice. MEF, middle ear fluid; DC, dendritic cell; NK, natural killer cell.

A similar analysis carried out on the *Fbxo11^tm2b/+^* heterozygote *Fbxo11* knockout mouse (**Table S3**), did not show the major differences in innate immune cell content as observed in the *Fbxo11^Jf/+^*mouse in comparison to their respective wild-type countertypes. The only significant difference observed for the *Fbxo11^tm2b/+^* compared to *Fbxo11^+/+^* mouse was for blood and lung macrophages and blood CD11b+ DC's. *Fbxo11^tm2b/+^* had a slight but significantly higher percentage of blood macrophages (1.72%) compared to the *Fbxo11^+/+^* mouse (0.95%). Also, CD11b+ DC's were 3.61% in *Fbxo11^tm2b/+^* mouse compared to 2.65% in the *Fbxo11^+/+^* mouse blood. In lungs of the *Fbxo11^tm2b/+^* mouse, macrophage numbers were significantly elevated (0.86%) compared to its wild-type counterpart, *Fbxo11^+/+^* (0.39%). We observed minor immune cell differences between the *Jeff* wild-type and *Fbxo11^+/+^* wild-type mice. It is known that mouse background affects immunity and the mouse immune response to infection. The *Jeff* wild-type and *Fbxo11^+/+^* mouse vary in their background: *Jeff* wild-type is on mixed C57BL/6J C3H/HeH background whereas the *Fbxo11^+/+^* mouse is on a C57BL/6NJ background, we predict that this difference in background could be influencing the difference in immune cell content observed between these wild-types.

Our observed differences in innate immune cells between the *Fbxo11^Jf/+^* and the *Fbxo11^tm2b/+^* mouse indicates the significant role played by the *Fbxo11* gene in immunogenetic control of these cells and OM.

### Adaptive Immune Cell Population in *Jeff* Mouse

**Figure 2:**
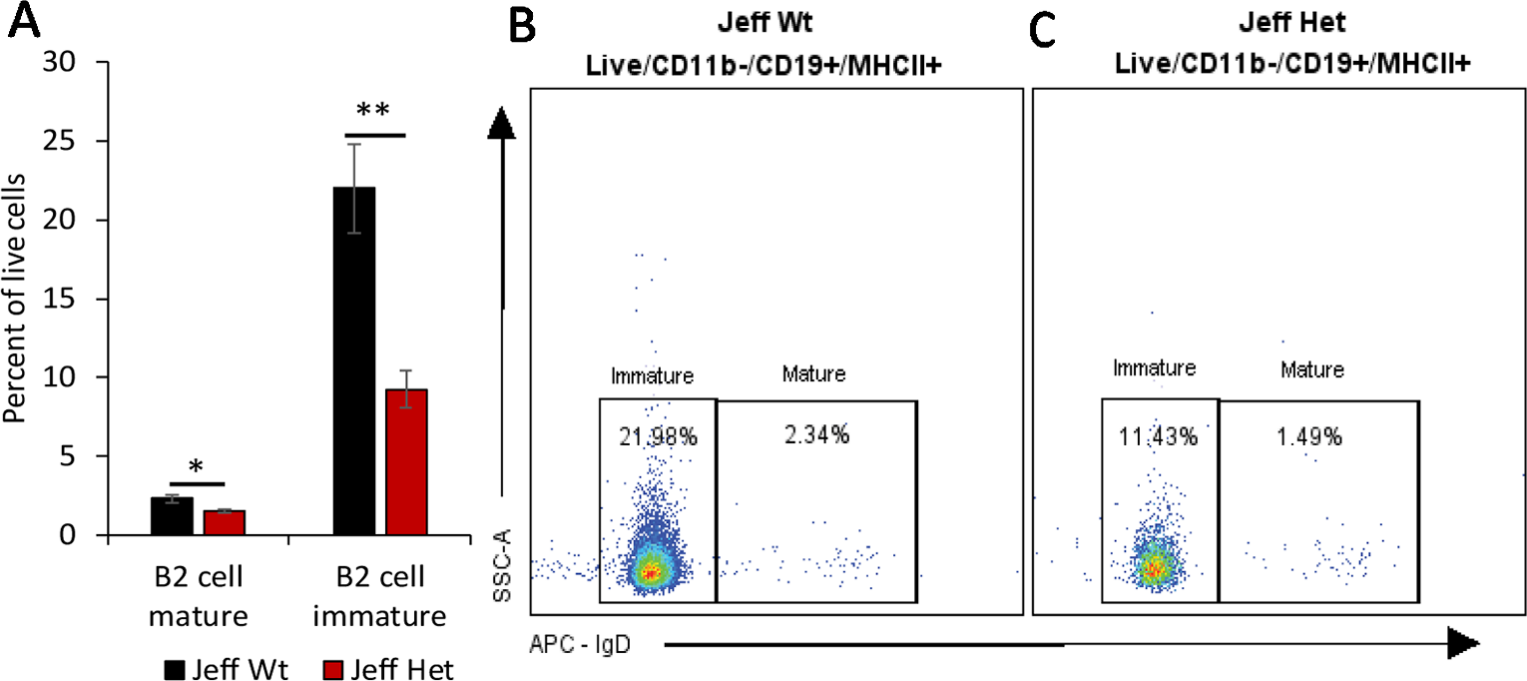
B cell percentage in *Jeff* mice (*Fbxo11^Jf/+^* and *Jeff* Wt). **(A)** Average difference in the percentage of mature and immature blood B2 cells in *Jeff* Het (*Fbxo11^Jf/+^*) and *Jeff* Wt mice. The error bars show standard error of mean where n = 4 and *P < 0.05, **P < 0.01. Representative flow cytometry plot for B cell analysis of bloods from *Jeff* Wt **(B)** and *Jeff* Het (*Fbxo11^Jf/+^*) **(C)** mice.

We characterized the content of adaptive immune cells (**Table 1**): T cells (helper and cytotoxic), B cells, and natural killer T (NKT) cells in *Fbxo11^Jf/+^* mouse blood, lung, spleen, and middle ear fluid. The percentage of T helper (Th) effector cells in the blood was significantly less in *Fbxo11^Jf/+^* (0.69%) compared to the *Jeff* wild-type counterpart. The percentage of blood resting T cytotoxic cells was also significantly less in the *Fbxo11^Jf/+^* (0.67%) compared to *Jeff* wild-type (1.56%) mouse. In contrast, the percentage of blood T regulatory (T reg) resting cells was significantly higher in the *Fbxo11^Jf/+^* compared to *Jeff* wild-type mouse. The greatest difference was observed in the proportion of blood immature B cells; *Fbxo11^Jf/+^* had significantly less (9.26%) immature B cells compared to the *Jeff* wild-type (21.98%) mouse (**Figure 2A–C**). In contrast, NKT cell levels in the blood were significantly higher in the *Fbxo11^Jf/+^* compared to *Jeff* wild-type mouse. *Fbxo11^Jf/+^* mouse blood had a significantly higher level of effector NKT cells, 2.04% compared to 0.77% in *Jeff* wild-type. A similar trend was observed in the levels of resting NKT cells and invariant NKT (iNKT) cells (**Figure 1D–F**, **Table 1**).

Analysis of cells derived from the lung did not show a significant difference in T-helper cells, but a significant reduction in the levels of naïve and resting T-cytotoxic cells was observed in the *Fbxo11^Jf/+^* compared to the *Jeff* wild-type mouse. The *Fbxo11^Jf/+^* lung had 0.58 and 0.1% of naïve and resting T cytotoxic cells respectively compared to 1.25 and 0.44% (naïve and resting respectively) in *Jeff* wild-type. Other than T-cytotoxic cells, the only significant reduction observed was in B2-immature cells; the *Fbxo11^Jf/+^* lung had 5.14% of B2-immature cells compared to 8.52% in the *Jeff* wild-type mouse. The percent of effector Th cells was significantly lower (0.72%) in *Fbxo11^Jf/+^* compared to 1.28% observed in the *Jeff* wild-type mouse whereas the resting T reg percentage was significantly higher in *Fbxo11^Jf/+^* spleen (1.34%) compared to that found in *Jeff* wild-type (0.82%). Also, a higher percentage of resting NKT cells was observed in *Fbxo11^Jf/+^* spleen (1.63%) compared to its wild-type counterpart (0.78%). In the middle ear fluid, the highest percentage of adaptive immune cells observed was NKT cells at 5.83%; among these NKT cells nearly 55% were effector cells. After NKT cells, B cells were the second highest adaptive immune cells observed in the *Fbxo11^Jf/+^* mouse middle ear fluid. The percentages of B1 and B2 cells in the *Fbxo11^Jf/+^* middle ear fluid were 1.74 and 1.37%, respectively and nearly 75% of the B2 cells were mature (**Table 1**).

Similar analysis carried out on the *Fbxo11^tm2b/+^* knockout mouse did not show major differences in adaptive immune cells as had been observed for the *Fbxo11^Jf/+^* mouse compared to the respective wild type (**Table S3**). Other than splenic immature B2 cells, no other significant differences were observed in the *Fbxo11^tm2b/+^* knockout compared to its wild-type counterpart *Fbxo11^+/+^*mouse. The *Fbxo11^tm2b/+^* mouse spleen had a significantly higher proportion of immature B2 cells (16.23%) compared to its wild-type counterpart *Fbxo11^+/+^* (8.60%). Our analysis indicates that the mutation in the *Fbxo11^Jf/+^* mouse affects not only the innate immune cell content but also adaptive immune cells. The absence of cellular immune changes in the *Fbxo11^tm2b/+^* mouse that were observed in the *Fbxo11^Jf/+^* mouse suggests that the ENU mutation in the *Fbxo11* gene could be gain of function which alters the regulation of immune cells, leading to lung and middle ear phenotypes observed in the *Fbxo11^Jf/+^* mice.

### Cytokine/Chemokine Levels in *Jeff* Mouse

**Figure 3:**
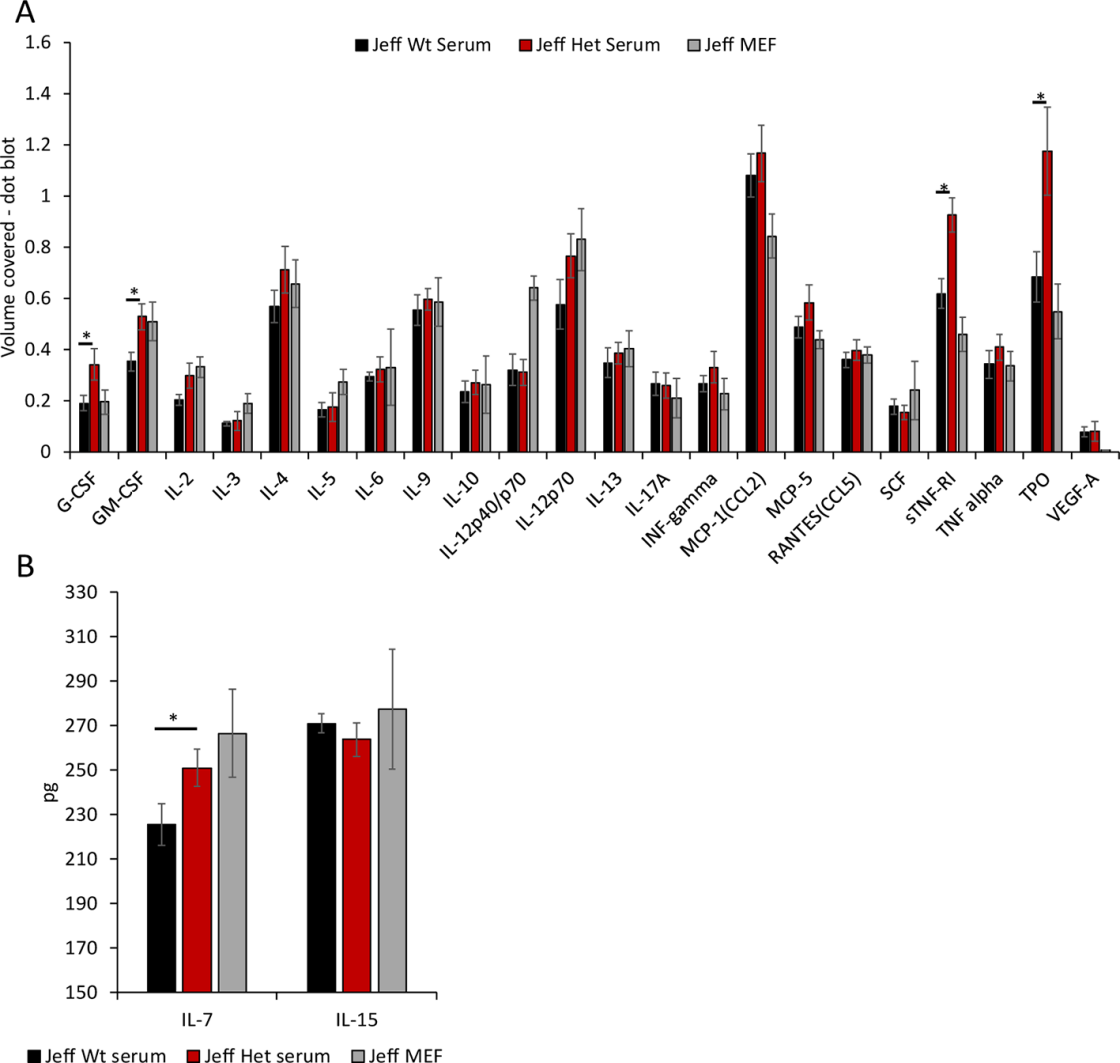
Cytokine and chemokine levels in *Jeff* mouse (*Fbxo11^Jf/+^* and *Jeff* Wt) serum and middle ear fluid (MEF). **(A)** The volume coverage represents the cytokine/chemokine levels obtained after analyses of 10 µl serum from *Jeff* Het (*Fbxo11^Jf/+^*) and *Jeff* Wt and 1 µl MEF from *Jeff* Het (*Fbxo11^Jf/+^*). Volume cover was calculated using Image Lab (BioRadTM) analysis on the RayBioRc C-Series mouse cytokine antibody array C1. **(B)** Interleukin (IL)-7 and IL-15 levels in the serum from *Jeff* Het (*Fbxo11^Jf/+^*) and *Jeff* Wt and MEF from *Jeff* Het (*Fbxo11^Jf/+^*), using mouse IL-7 or IL-15 DuoSet ELISA (R&D systems™). The concentrations in pg/ml were obtained from the standard graph of concentration against absorbance at 450 nm. The error bars show standard error of mean where n = 4 and *P < 0.05.

Mutation in the *Fbxo11* gene leads to systemic and local differences in the *Fbxo11^Jf/+^* mouse immune cells. To understand the influence of the autocrine and paracrine signals on the differences observed, levels of the specific cytokines/chemokines that modulate the functional response in the immune cells were analyzed (**Figure 3A**). A significant increase in G-CSF, GM-CSF, sTNFRI (soluble TNFRI), and TPO levels was observed in the *Fbxo11^Jf/+^* serum compared to the *Jeff* wild-type. The significant increase in the NK cells observed in the *Fbxo11^Jf/+^* mouse could correlate with the major cytokines known to modulate NK cell number and function, IL-7 and IL-15 (**Figure 3B**). Of these two cytokines, only IL-7 was significantly higher in *Fbxo11^Jf/+^* serum compared to its wild-type counterpart mouse. A majority of the cytokine/chemokines in the *Fbxo11^Jf/+^* mouse middle ear fluid were relatively high: IL-12p40/p70, IL-12p70, CCL2, sTNFRI, and TPO. High levels of IL-7 were also observed in the *Fbxo11^Jf/+^* middle ear fluid.

## Discussion

One of the best characterized animal models for chronic OM is the *Jeff* mouse mutant, which carries a mutation in the *Fbxo11* gene that is known to be associated with human OM (Hardisty et al., 2003; Segade et al., 2006; Bhutta et al., 2017a). To understand the effect of this mutation on the systemic and localized immune cell content, we analyzed the immune cell population in the *Jeff* mutant mouse.

The most significant difference observed between *Jeff* heterozygotes (*Fbxo11^Jf/+^*) and wild-type mice was in the increased percentage of NK cells in blood, which are significantly raised in the mutant. Similarly, the percentage of NKT cells (effector, resting, and iNKT) was high in the *Fbxo11^Jf/+^* compared to the wild-type mouse. Analysis carried out on otitis-prone children has shown similar findings; children prone to OM have a higher proportion of NK cells in blood compared to a healthy control group. The authors of the study postulate that the increase in NK cell numbers is due to prolonged exposure to upper respiratory tract infection (Seppanen et al., 2018). However, our current study provides the possibility of a genetic link being responsible for the increase in the circulating NK cells. The *Fbxo11^Jf/+^* mice utilized in the present study are bred in specific pathogen-free conditions (Hood et al., 2016). The development of OM in *Fbxo11^Jf/+^* mice is spontaneous at a young age (Hardisty et al., 2003). A similar analysis carried out in the *Fbxo11^tm2b/+^* knockout mouse did not show any difference in the NK cell population when compared to its wild-type counterpart. This indicates that the increase in the NK cells in the circulation can be purely attributed to the specific mutation in the *Fbxo11* gene in the *Fbxo11^Jf/+^* mouse.

One of the major cytokines that plays a critical role in NK cell development, homeostasis, and activation is IL-15 (Marçais et al., 2013). Mice deficient in IL-15 do not produce NK cells, supporting its significance in NK cell development (Ranson et al., 2003). Analysis of IL-15 levels in the *Fbxo11^Jf/+^* mouse serum did not show a significant difference compared to its wild-type counterpart indicating that the *Fbxo11* mutation does not affect the paracrine signaling for NK cell modulation through IL-15. IL-15 maintains the peripheral NK cell numbers by increasing the anti-apoptotic BCL6 family proteins (Ranson et al., 2003). BCL6 is targeted for ubiquitination and proteasomal degradation by an SKP1–CUL1–F- box protein (SCF) ubiquitin ligase complex that contains the FBXO11 protein (Duan et al., 2012). The increased blood NK cell percentage in the *Fbxo11^Jf/+^* mouse indicates the possible alteration of the SCF mediated BCL6 degradation, specifically in the NK cells. Another important cytokine that regulates NK cell function is TGF-beta, levels of which were not significantly different in the *Fbxo11^Jf/+^* mouse compared to its wild-type counterpart. Earlier studies carried out on the *Fbxo11^Jf/+^* mouse have highlighted the significance of the *Jeff* mutation on the Smad2 mediated TGF-beta induced intracellular signaling in the developmental pathways (Tateossian et al., 2009). Even though we did not observe any significant difference in the levels of TGF-beta in the *Fbxo11^Jf/+^* mouse, the intracellular signaling induced by TGF-beta in the NK cells could play a vital role in their increased levels and function.

Along with NK cells, we also observed a significant increase in the circulating T cell population in the *Fbxo11^Jf/+^*mouse. One of the cytokines that can stimulate the release of lymphocytes from bone marrow and thymus is IL-7 (von Freeden-Jeffry et al., 1995); levels of this cytokine were significantly higher in the *Fbxo11^Jf/+^* serum compared to its wild-type counterpart. Along with increasing lymphocyte numbers, this elevated level of IL-7 can also result in increased thymic NK cell numbers (Marçais et al., 2013). IL-7 also increases the release of B cells from bone marrow and thymus (von Freeden-Jeffry et al., 1995) but in *Fbxo11^Jf/+^* the percentage of B cells was significantly lower than in the wild-type mouse. FBXO11 is known to play a critical role in maintenance of B cell levels *via* the BCL6 pathway and FBXO11 loss of function is frequently observed in B cell lymphoma cell lines and increased B-cell levels (Duan et al., 2012), as we observed in the *Fbxo11^tmb2/+^* knockout mouse. Restoration of BCL6 function in the B cell lymphoma cell lines has been shown to inhibit B-cell lymphogensis (Duan et al., 2012), indicating a potential critical role played by FBXO11 in the modulation of B cell survival. Our observation of reduced levels of B cells in the *Fbxo11^Jf/+^* mouse indicates that the *Jeff* mutation in the B-cells could be inducing the apoptotic pathways, possibly through BCL6. This novel role of *Fbxo11* in the modulation of B cell survival warrants further studies but suggest that the *Jeff* mutation may be manifesting gain of function phenotypes in the regulation of B cell numbers.

The other cytokines that were significantly increased in the *Fbxo11^Jf/+^* serum compared to wild-type were G-CSF and GM-CSF. Both these cytokines play a critical role in the regulation of neutrophil development (Roberts, 2005), which could correspond with the significantly higher blood granulocyte levels observed in the *Fbxo11^Jf/+^* mouse. G-CSF also inhibits NK cell activity (Schlahsa et al., 2011). IL-2 is one of the major cytokines released after activation of NK cells. In the *Fbxo11^Jf/+^* mouse, even with the high percentage of NK cells in the circulation, no difference in IL-2 levels was observed compared to wild type. Neutrophil numbers also play a critical role in perturbation of inflammation in the middle ear. In other animal models, the extracellular neutrophil DNA in the middle ear fluid contributes to NTHi biofilm formation (Jurcisek and Bakaletz, 2007; Juneau et al., 2011). In the case of *Fbxo11^Jf/+^* mice, the neutrophil percentage in the middle ear fluid was high and most of these were living. This might be a contributing factor toward the significantly lower NTHi titers and infection rate observed in *Fbxo11^Jf/+^* compared to *Junbo* mice (Hood et al., 2016); the latter has high levels of necrotic cells in the middle ear fluid (Vikhe et al., 2019).

The level of soluble TNFRI (sTNFR1) in the *Fbxo11^Jf/+^* serum was significantly higher than that found in wild-type, whereas TNF–alpha levels were not different. sTNFRI has a high affinity for TNF-alpha (Wang et al., 2004), modulating its slow release and thus suppressing inflammation. It is well established that inhibiting TNF-alpha decreases middle ear inflammation in OM (Jeun et al., 2001). Trans-tympanic membrane treatment of mice with sTNFRI and anti-TNF-alpha inhibits LPS induced middle ear inflammation and OM (Jeun et al., 2001). The molecular level regulation for the release of sTNFRI is still unclear. The increase in sTNFRI in the *Fbxo11^Jf/+^* mouse serum indicates a role for FBXO11 in its regulation; this mouse model could play an essential role in understanding the regulation of this critical cytokine at the genetic level.

Another cytokine/growth factor that was significantly higher in *Fbxo11^Jf/+^* mouse serum was thrombopoietin (TPO). TPO is a growth factor that initiates the process of megakaryocyte production and generation of platelets (Lupia et al., 2012). Increased levels of TPO are found in patients with inflammatory bowel disease (Kapsoritakis et al., 2000) and *Streptococcus pneumoniae* induced bacterial meningitis in mice (Hoffmann et al., 2011). The relevance of TPO in cardiovascular damage and inflammatory diseases is known (Lupia et al., 2012), but its role in OM has yet to be evaluated. We were not able to classify platelet population in the *Fbxo11^Jf/+^* mouse in our current study but increased levels of TPO are indicative of abnormal platelet function and its relevance in OM can be investigated using this mutant mouse model.

In conclusion, our studies of the *Jeff* (*Fbxo11^Jf/+^*) mouse model identify immune cell changes which can play a critical role in inflammation. A significant increase in blood neutrophil, NK cells, NKT cells and dendritic cells were observed in the *Fbxo11^Jf/+^* mutant mouse compared to wild-type. The heterozygote *Fbxo11* knockout mouse did not show these differences, consistent with a gain of function for the *Jeff* mutation that leads to these immune cell changes. Interestingly, as we report elsewhere (Kubinyecz *et al*. manuscript submitted), the *Fbxo11^tm2b/+^* heterozygous knockout mouse does not develop a spontaneous OM phenotype as observed in *Fbxo11^Jf/+^*, which is also indicative of gain of function effects. In conclusion, this study paves the way for further studies using the *Jeff* model to dissect the genetic interrelationships between the control of immune cells and OM.

## Data Availability Statement

All datasets generated for this study are included in the article/**Supplementary Material**.

## Ethics Statement

The animal study was reviewed and approved by MRC Harwell Institute ethics review committee and this study was carried out under the appropriate UK Home Office license.

## Author Contributions

PV designed and conceptualized the experiments. PV, HT, and GB performed the experiments. PV, and GB analyzed the data. PV and HT participated in collecting animals testes. PV drafted the manuscript. PV, HT, DH, and SB edited and wrote the final manuscript. All authors approved the manuscript for submission.

## Funding

This work was funded by the Medical Research Council (MRC funding award no. MC_U142684175).

## Conflict of Interest

The authors declare that the research was conducted in the absence of any commercial or financial relationships that could be construed as a potential conflict of interest.

## Supplementary Material

Figure S1 Flow cytometry panel 1 gating strategy.

Figure S2 Flow cytometry panel 2 gating strategy.

Table S1 Flow cytometry antibody and panels.

Table S2 Flow cytometry gating strategy.

Table S3 Immune cell percentage in *Fbxo11^tmb2/+^* (*Fbxo11* knockout) and *Fbxo11^+/+^* mouse obtained by flow cytometry.
