# Supplementary figures and images for: Mutation in Fbxo11 Leads to Altered Immune Cell Content in Jeff Mouse Model of Otitis Media
Source: Front Genet. 2020 Feb 11;11:50. doi: 10.3389/fgene.2020.00050 (PMC7026503; doi:10.3389/fgene.2020.00050)

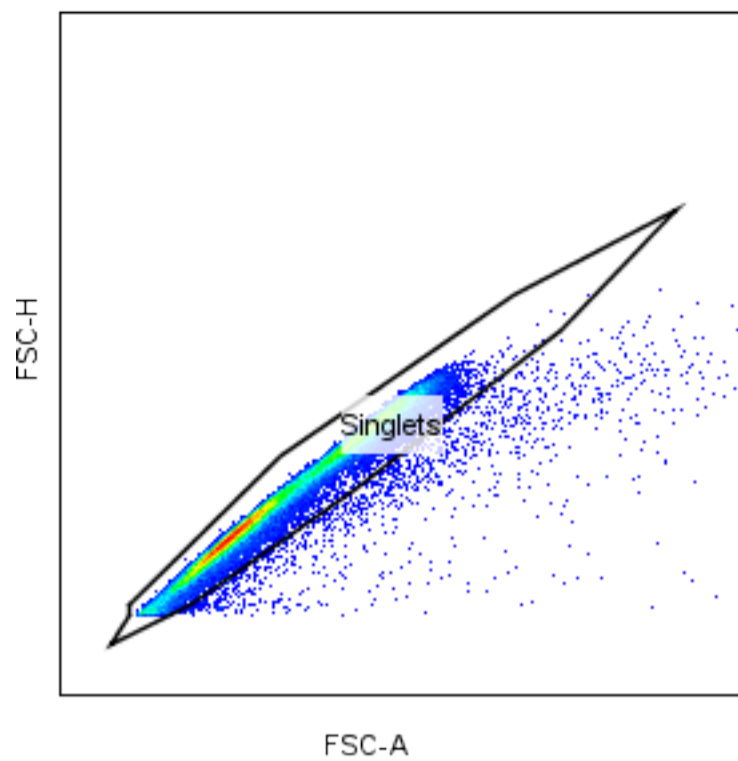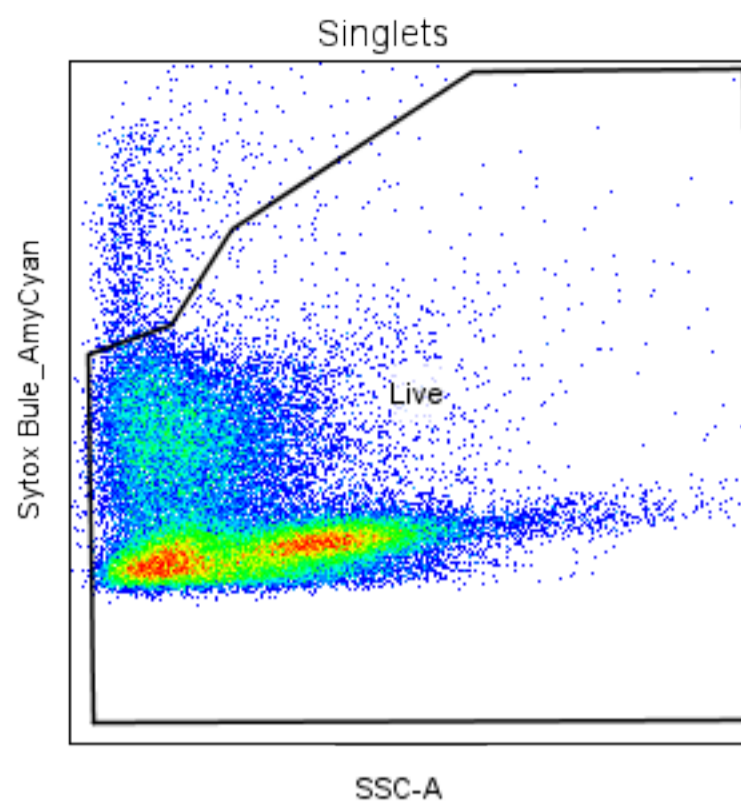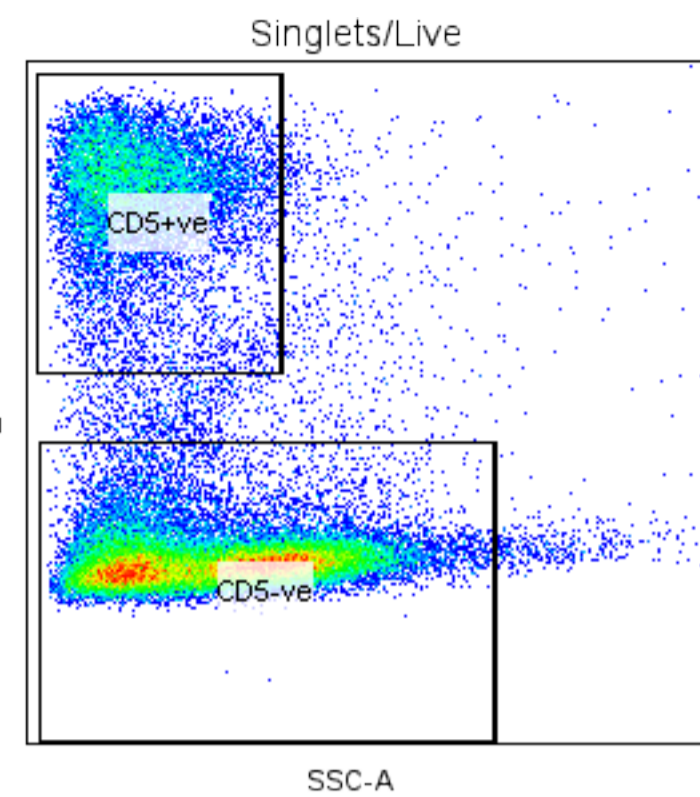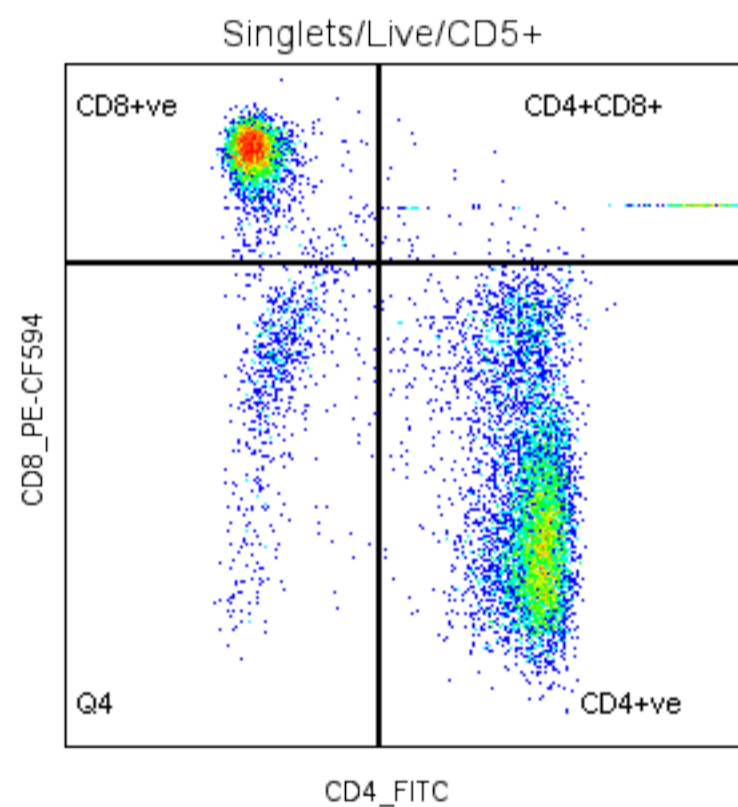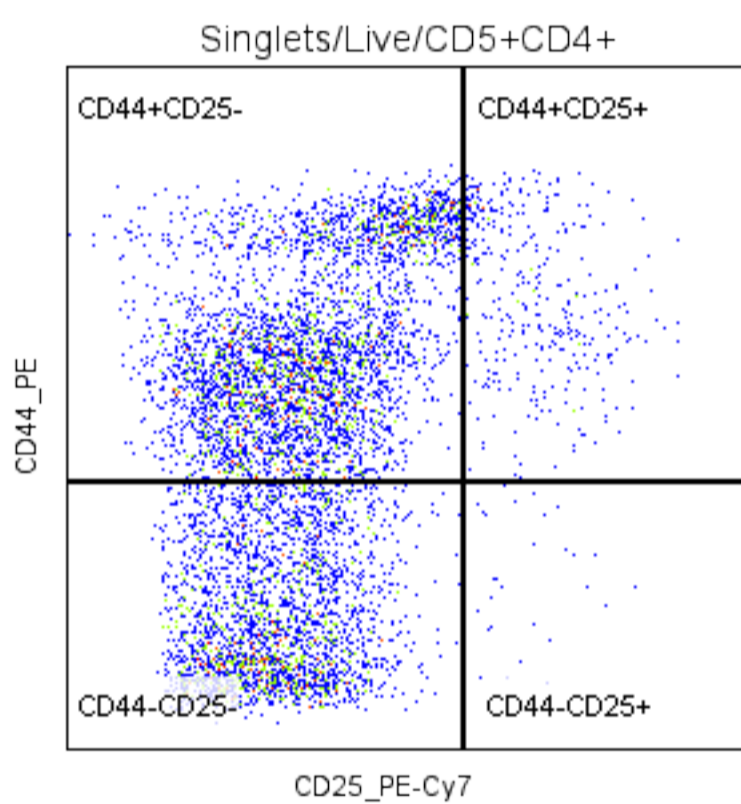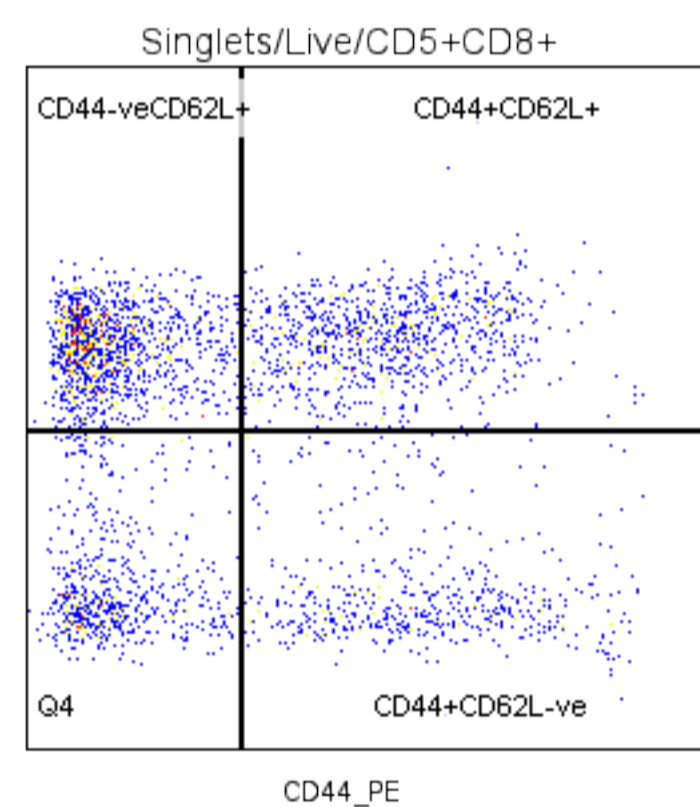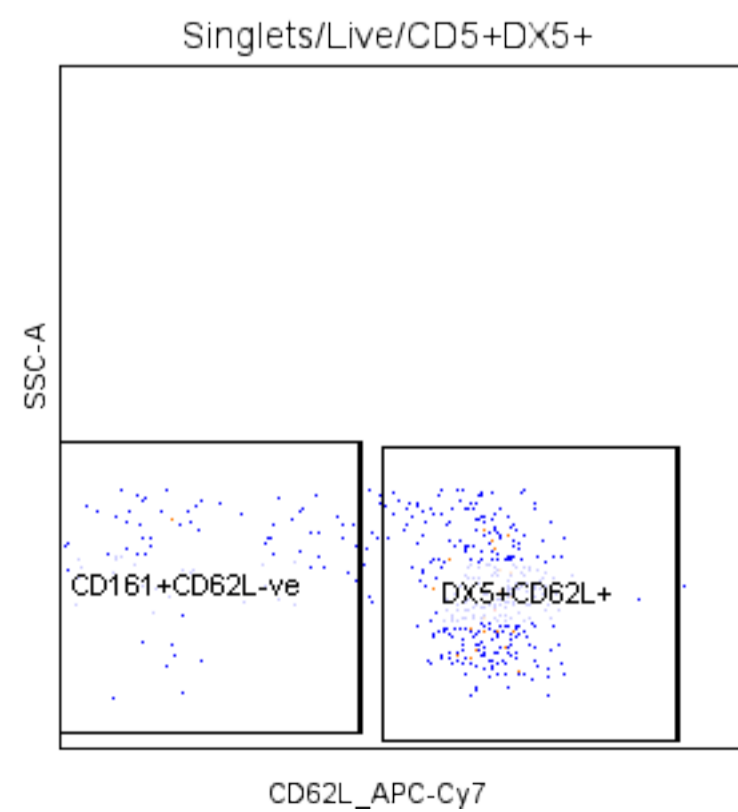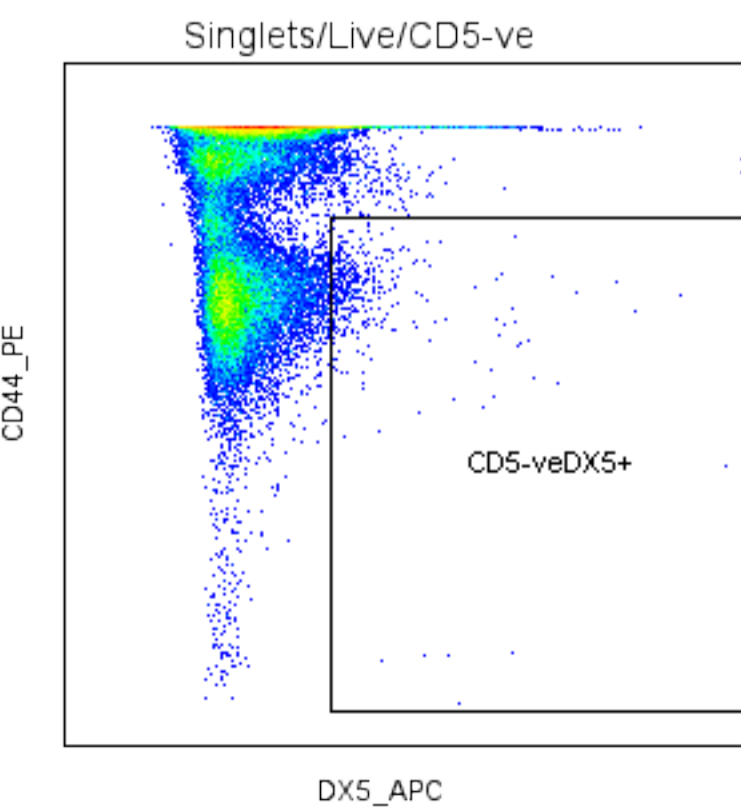

Supplement: Figure S1 — Flow cytometry panel 1 gating strategy. [file Image_1.pdf]

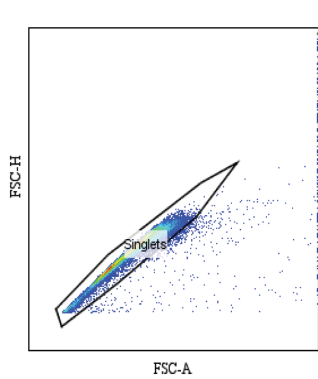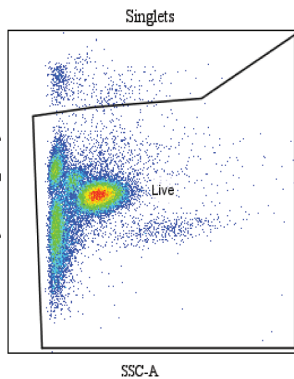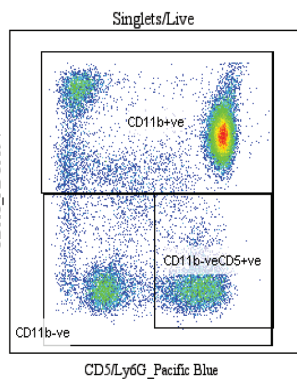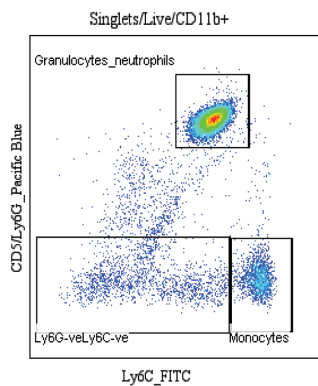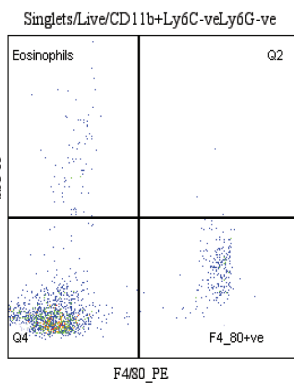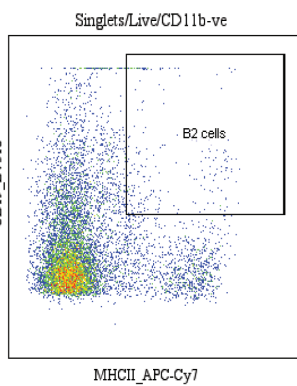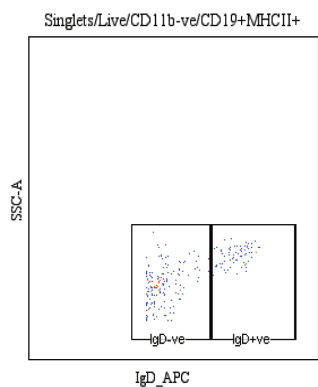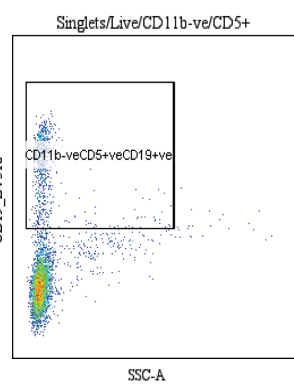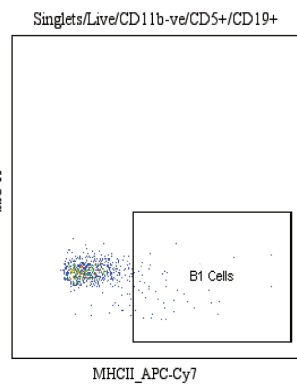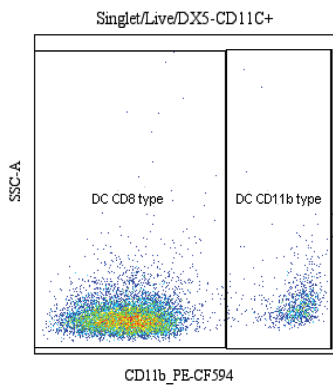

Supplement: Figure S2 — Flow cytometry panel 2 gating strategy. [file Image_2.pdf]
