# Supplementary material for: Mutation in Fbxo11 Leads to Altered Immune Cell Content in Jeff Mouse Model of Otitis Media
Source: Front Genet. 2020 Feb 11;11:50. doi: 10.3389/fgene.2020.00050 (PMC7026503; doi:10.3389/fgene.2020.00050)
Supplement: Table S1 — Flow cytometry antibody and panels. [file Table_1.pdf]

Panel 1:				
**Marker / Antigen**	**Fluorophore**	**Concentration**	**Source**	**Cat no.**
Live/dead	SytoxBlue	1:10000	Invitrogen	S11348
CD5	BV421	1:200	BD biosciences	562739
CD4	FITC	1:800	BD biosciences	553047
CD8	PE-CF594	1:400	BD biosciences	562283
CD25	PECy7	1:400	BD biosciences	552880
CD62L	APC-Cy7	1:1600	BD biosciences	560514
CD44	PE	1:200	BD biosciences	553134
TCRd	BV510	1:100	BD biosciences	563218
DX5 for *Jeff* mice or NK1.1 for *Fbxo11*$^{tmb2/+}$	APC	1:200	BD biosciences	560628 (DX5) 550627(NK1.1)

Panel 2:				
**Marker / Antigen**	**Fluorophore**	**Concentration**	**Source**	**Cat no.**
Live/dead	SytoxBlue	1:10000	Invitrogen	S11348
F4/80	PE	1:400	BD biosciences	552957
CD19	BV510	1:400	BD biosciences	562956
DX5 for *Jeff* mice or NK1.1 for *Fbxo11*$^{tmb2/+}$	APC	1:200	BD biosciences	560628 (DX5) 550627(NK1.1)
Ly6C	FITC	1:400	BD biosciences	553104
Ly6G	BV421	1:400	BD biosciences	562737
CD5	BV421	1:200	BD biosciences	562739
CD11b	PE-CF594	1:4800	BD biosciences	562287
CD11c	PECy7	1:400	BD biosciences	558079
IgD	APC	1:200	BD biosciences	560868
MHCII (anti-Mouse I-A/I-E)	APC-Cy7	1:200	BioLegend	107628
