# Supplementary material for: Mutation in Fbxo11 Leads to Altered Immune Cell Content in Jeff Mouse Model of Otitis Media
Source: Front Genet. 2020 Feb 11;11:50. doi: 10.3389/fgene.2020.00050 (PMC7026503; doi:10.3389/fgene.2020.00050)
Supplement: Table S2 — Flow cytometry gating strategy. [file Table_2.pdf]

Gating strategy employed for classification of immune cells		
**Cells**	**Positive Markers**	**Negative markers**
Granulocytes/neutrophils	CD11b+Ly6G+Ly6C+	Sytox blue
Eosinophils	CD11b+SSC-Ahi	Sytox blue/Ly6C-Ly6G-
Macrophages	CD11b+SSClowF4/80+	Sytox blue/Ly6C-Ly6G-
Monocytes	CD11b+Ly6C+	Sytox blue/Ly6G-
Dendritic cells_CD8 type	CD11C+MHCII+	Sytox blue/DX5-CD11b-
Dendritic cells_CD11b type	CD11C+MHCII+CD11b+	Sytox blue/DX5-
Natural killer (NK) cells	DX5+	Sytox blue/CD5-
T helper (Th)	CD5+CD4+	Sytox blue/CD8-
T helper effector	CD5+CD4+CD44+	Sytox blue/CD8-
T regulatory effector	CD5+CD4+CD25+CD44+	Sytox blue/CD8-
T regulatory	CD5+CD4+CD25+	Sytox blue/CD8-
T cytotoxic (Tcyto)	CD5+CD8+	Sytox blue/CD4-
T cytotoxic effector	CD5+CD8+CD44+	Sytox blue/CD4-CD62L-
T cytotoxic naive	CD5+CD8+CD62L+	Sytox blue/CD4-CD44-
T cytotoxic resting	CD5+CD8+CD62L+CD44+	Sytox blue/CD4-
B2 mature cells	CD19+MHCII+IgD+	Sytox blue/CD11b-CD5-
B2 immature cells	CD19+MHCII+	Sytox blue/CD11b-CD5-IgD-
B1 cells	CD5+CD19+MHCII+	Sytox blue/CD11b-
NK-T cells	CD5+CD44+DX5+	Sytox blue
NK-T cell effector	CD5+CD44+DX5+	Sytox blue/CD62L-
NK-T cell resting	CD5+CD44+DX5+CD62L+	Sytox blue
