# Supplementary material for: Mutation in Fbxo11 Leads to Altered Immune Cell Content in Jeff Mouse Model of Otitis Media
Source: Front Genet. 2020 Feb 11;11:50. doi: 10.3389/fgene.2020.00050 (PMC7026503; doi:10.3389/fgene.2020.00050)
Supplement: Table S3 — Immune cell percentage in Fbxo11tmb2/+ (Fbxo11 knockout) and Fbxo11+/+ mouse obtained by flow cytometry. [file Table_3.pdf]

Table S3: Immune cell percentage in *Fbxo11*$^{+/+}$ and *Fbxo11*$^{tmb2/+}$ mouse obtained by flow cytometry.

Immune cells	Blood		Lungs		Spleen	
	Fbxo11$^{+/+}$	*Fbxo11*$^{tmb2/+}$	*Fbxo11*$^{+/+}$	*Fbxo11*$^{tmb2/+}$	*Fbxo11*$^{+/+}$	*Fbxo11*$^{tmb2/+}$
**Granulocytes**	11.99 (±1.01)	15.54 (±1.62)	6.54 (±0.79)	7.88 (±1.06)	1.81 (±0.43)	3.52 (±1.39)
**Eosinophils**	1.02 (±0.39)	0.82 (±0.45)	0.02 (±0.01)	0.02 (±0.002)	0.04 (±0.01)	0.038 (±0.02)
**Macrophages**	0.95 (±0.09)	1.72 (±0.17)	0.39 (±0.04)	0.86 (±0.07)	0.39 (±0.06)	0.51 (±0.06)
**Monocytes**	6.66 (±0.69)	7.97 (±1.36)	1.43 (±0.21)	2.19 (±0.64)	0.94 (±0.07)	1.64 (±0.50)
**DC CD8 type**	38.54 (±3.39)	29.66 (±5.37)	12.1 (±1.52)	13.4 (±1.30)	55.92 (±0.56)	52.04 (±1.30)
**DC CD11b type**	2.65 (±0.17)	3.61 (±0.22)	1.87 (±0.15)	2.28 (±0.22)	4.14 (±0.67)	3.95 (±0.80)
**Progenitor DC (pDC)**	3.36 (±0.64)	2.57 (±0.69)	13.50 (±0.89)	13.30 (±0.62)	1.66 (±0.54)	2.26 (±0.41)
**NK cells**	6.92 (±0.36)	5.80 (±0.27)	1.45 (±0.17)	1.89 (±0.27)	3.64 (±0.32)	2.37 (±0.21)
**T helper (Th) Effector**	1.26 (±0.27)	1.53 (±0.09)	0.36 (±0.13)	0.23 (±0.04)	0.47 (±0.08)	0.42 (±0.05)
**T helper (Th) Resting**	2.42 (±0.26)	2.60 (±0.13)	1.65 (±0.50)	1.27 (±0.05)	4.67 (±1.28)	3.61 (±0.71)
**T regulator (T reg) Effector**	0.54 (±0.07)	0.67 (±0.03)	0.32 (±0.12)	0.33 (±0.008)	0.54 (±0.08)	0.65 (±0.10)
**T regulator (T reg) Resting**	0.29 (±0.06)	0.35 (±0.08)	0.43 (±0.06)	0.38 (±0.04)	2.89 (±1.47)	4.22 (±1.17)
**T cytotoxic Effector**	0.66 (±0.07)	0.72 (±0.06)	0.22 (±0.10)	0.34 (±0.05)	0.59 (±0.09)	0.54 (±0.04)
**T cytotoxic Naive**	2.52 (±0.22)	2.83 (±0.37)	1.08 (±0.11)	0.96 (±0.05)	5.28 (±0.35)	4.81 (±0.14)
**T cytotoxic Resting**	1.63 (±0.24)	2.19 (±0.48)	0.38 (±0.07)	0.41 (±0.03)	3.90 (±0.35)	4.13 (±0.64)
**B2 total**	19.21 (±5.02)	20.33 (±4.38)	11.10 (±1.09)	9.54 (±0.48)	10.13 (±4.89)	18.94 (±2.25)
**B2 mature**	1.17 (±0.52)	1.06 (±0.42)	2.26 (±0.48)	2.3 (±0.05)	1.26 (±0.82)	2.13 (±0.86)
**B2 immature**	18.03 (±4.36)	19.27 (±3.85)	8.57 (±0.85)	7.02 (±0.55)	8.60 (±1.84)	16.23 (±2.18)
**B1 total**	0.71 (±0.21)	0.95 (±0.32)	8.68 (±0.48)	8.97 (±0.21)	2.85 (±1.63)	4.51 (±1.61)
**NKT Effector**	2.98 (±0.66)	3.37 (±0.42)	14 (±0.88)	17.46 (±1.71)	1.45 (±0.11)	1.92 (±0.11)
**Invariant NKT (iNKTs)**	2.99 (±0.91)	3.26 (±0.57)	7.08 (±1.91)	9.75 (±1.32)	1.46 (±0.12)	1.91 (±0.15)
**NKT Resting**	2.03 (±0.88)	1.98 (±0.58)	1.69 (±0.11)	1.40 (±0.12)	1.61 (±0.15)	1.93 (±0.25)

Values are the percentage of total live cells. Underlined values are significantly increased whereas those in italics are significantly decreased in *Fbxo11*$^{+/+}$ compared to *Fbxo11*$^{tmb2/+}$ mice. MEF – middle ear fluid, DC – Dendritic cell, NK- Natural killer cell.
